# Construction of a high-density genetic map for hexaploid kiwifruit (*Actinidia chinensis* var. *deliciosa*) using genotyping by sequencing

**DOI:** 10.1093/g3journal/jkab142

**Published:** 2021-05-01

**Authors:** Elizabeth Popowski, Susan J Thomson, Mareike Knäbel, Jibran Tahir, Ross N Crowhurst, Marcus Davy, Toshi M Foster, Robert J Schaffer, D Stuart Tustin, Andrew C Allan, John McCallum, David Chagné

**Affiliations:** 1 The New Zealand Institute for Plant and Food Research Ltd (Plant & Food Research), Te Puke, New Zealand; 2 Plant & Food Research, Lincoln, New Zealand; 3 Plant & Food Research, Palmerston North, New Zealand; 4 Plant & Food Research, Auckland, New Zealand; 5 Plant & Food Research, Motueka, New Zealand; 6 School of Biological Sciences, University of Auckland, Auckland, New Zealand; 7 Plant & Food Research, Havelock North, New Zealand

**Keywords:** polyploid, SNP markers, linkage mapping, QTLs, Ericaceae

## Abstract

Commercially grown kiwifruit (genus *Actinidia*) are generally of two sub-species which have a base haploid genome of 29 chromosomes. The yellow-fleshed *Actinidia chinensis* var. *chinensis*, is either diploid (*2n *=* 2x *=* *58) or tetraploid (*2n *=* 4x *=* *116) and the green-fleshed cultivar *A. chinensis* var. *deliciosa* “Hayward,” is hexaploid (*2n *=* 6x *=* *174). Advances in breeding green kiwifruit could be greatly sped up by the use of molecular resources for more efficient and faster selection, for example using marker-assisted selection (MAS). The key genetic marker that has been implemented for MAS in hexaploid kiwifruit is for gender testing. The limited marker-trait association has been reported for other polyploid kiwifruit for fruit and production traits. We have constructed a high-density linkage map for hexaploid green kiwifruit using genotyping-by-sequence (GBS). The linkage map obtained consists of 3686 and 3940 markers organized in 183 and 176 linkage groups for the female and male parents, respectively. Both parental linkage maps are co-linear with the *A. chinensis* “Red5” reference genome of kiwifruit. The linkage map was then used for quantitative trait locus (QTL) mapping, and successfully identified QTLs for king flower number, fruit number and weight, dry matter accumulation, and storage firmness. These are the first QTLs to be reported and discovered for complex traits in hexaploid kiwifruit.

## Introduction

Originating in China and Southeast Asia, kiwifruit (genus *Actinidia*) has been described as a woody, perennial, deciduous vine, producing cylindrical fleshy fruit (Schmid 1978). In the last 60 years, kiwifruit has become the largest horticultural export item for New Zealand (Ferguson 2004) generating NZD$2.3 billion in exports in 2019 (https://www.freshfacts.co.nz/). The two most commonly exported species are *Actinidia chinensis* var. *deliciosa* and *A. chinensis* var. *chinensis*. Outside of China, the main *A. chinensis* var. *deliciosa* cultivar is green-fleshed “Hayward,” accounting for 80% of global kiwifruit production (Ferguson 2015). The genus *Actinidia* is very diverse in terms of morphological and genetic variation (Ferguson and Huang 2007). Naturally occurring ploidy levels are diploid, tetraploid, hexaploid, and octoploid (Guijun *et al.* 1994). The *A. chinensis* var. *deliciosa* genotypes (green-fleshed kiwifruit) from the New Zealand breeding program are hexaploid with six sets of 29 chromosomes (*2n *=* 6x *=* *174) (Watanabe *et al.* 1990).

Polyploid species can be classified as allo- and autopolyploid. An allopolyploid is described as being comprised of sub-genomes originating from multiple related species and displaying chromosomal preferential pairing during meiosis. Notable allopolyploid crop species include hexaploid bread wheat (*Triticum aestivum*) and tetraploid canola (*Brassica napus*). By contrast, an autopolyploid is created during a genome duplication event and in general does not display preferential chromosome pairing. An analysis of *A. chinensis* var. *deliciosa* using restricted fragment length polymorphism (RFLP) markers proposed *A. chinensis* var. *chinensis* as one progenitor for hexaploid kiwifruit (Atkinson *et al.* 1997). However, additional progenitors contributing to at least two sets of homologs are unknown. Microsatellite markers used to track the inheritance of alleles in a hybrid population (*A. chinensis* var. *deliciosa* crossed with *A. chinensis* var. *eriantha*) suggested there is no preferential pairing between the chromosome homologs (Mertten *et al.* 2012). Therefore, *A. chinensis* var. *deliciosa* could be classified as an allopolyploid due to its homeologous sub-genomes obtained from several species, but functionally pairing as an autopolyploid. Phenotypically, a significant increase in fruit weight is observed as the ploidy level increases (Li *et al.* 2013). This has been confirmed with the genome doubling of diploid kiwifruit to autotetraploid using colchicine (Wu *et al.* 2012).

Reference genome assemblies have been developed for diploid *A. chinensis* var. *chinensis* (Huang *et al.* 2013; Pilkington *et al.* 2018; Wu *et al.* 2019), *A. eriantha* (Tang *et al.* 2019), and *A. rufa* (Zhang *et al.* 2015). Both the *A. eriantha* and *A. chinensis* genome v3.0 (Wu *et al.* 2019) were based on long read sequencing technologies incorporating high-throughput chromatin capture (Hi-C) methodologies with each genome assembly being assigned to the 29 pseudo-chromosomes. An assembly of a diploid *A. chinensis* selection (Pilkington *et al.* 2018), known as Red5, presents an improvement in terms of percentage of the assembly anchored to pseudo-chromosomes (only 6 Mb of the assembly were not assigned to chromosomes) and an improved gene annotation compared to the first draft assembly obtained from the cultivar *A. chinensis* Hongyang: [164 Mb unassigned to chromosomes (Huang *et al.* 2013)]. Linkage maps have been constructed for diploid *Actinidia* (Testolin *et al.* 2001; Fraser *et al.* 2009; Scaglione *et al.* 2015; Liu *et al.* 2017) using a range of molecular markers including amplified fragment length polymorphisms (AFLPs), simple sequence repeats (SSRs), and genotyping by sequencing (GBS) (Elshire *et al.* 2011). GBS has recently been the method of choice for building linkage maps in diploid kiwifruit, including RAD-seq (Scaglione *et al.* 2015; Liu *et al.* 2017), GBS (Tahir *et al.* 2019), and hybridization-based (Liu *et al.* 2016) approaches. The advantage of GBS is that this method can provide the sequence and haplotype data while generating the number of markers to sufficiently saturate all the homologs from a complex ploidy species.

Due to *A. chinensis* var. *deliciosa* requiring 3–5 years (Testolin *et al.* 2016) to establish a canopy before flower/fruit development occurs, advances in breeding green kiwifruit are constrained by the long generation time. Marker-assisted selection (MAS) would facilitate a more efficient and faster selections, but the high ploidy makes this a considerable challenge with only dominant traits such as gender testing (Gill *et al.* 1998) and the recently developed vitamin C marker being deployed (McCallum *et al.* 2019). The objective of this study was to construct a high-density linkage map for hexaploid green kiwifruit (*A. chinensis* var. *deliciosa*) using GBS to allow more complex traits to be mapped and selected for using MAS in the future.

## Materials and methods

### Plant material

A F1 population from a bi-parental (*A. chinensis* var. *deliciosa*) cross between a hexaploid female ZE and hexaploid male 28 was used in this study (Supplementary Figure S1). Kiwifruit is dioecious, the male and female reproductive organs are on separate plants, which promotes outcrossing (allogamy). The cross was completed in November 2007 with the seeds from the resulting ZE28 family extracted in April 2008. To test the relatedness of the parents, the coefficient of coancestry (*f_XY_*) was calculated using the pedigree structure (Bernardo 2002) and *f_XY_* = 0.

The population was generated by germinating seedlings on which MAS gender testing was performed (Gill *et al.* 1998) and used to identify and reduce the male proportion prior to planting in the orchard. Thousand one hundred and sixty-eight seedlings were planted across four different trial blocks at the Plant & Food Research, Te Puke Research Center (Table 1) of which 86% of these were female. Blocks 3 and 52 were planted at a density of 10 plants per 6 m bay, with 4 m between rows (4167 plants/Ha) while blocks 14 W and 35 were planted with 14 plants (seven plants on each side of the row) at a density of seven plants in a 6 m bay with a row spacing of 5 m (4667 plants/Ha). Males or unidentified gender plants were at a density of one in each bay to ensure adequate pollination. Each seedling planted was a unique genotype.

**Table 1:** The total number of ZE28 seedlings planted and the subset of quantitative trait loci (QTL) analysis, including the transplanting date, by the block, planting date, and number of females (♀ ), males (♂ ), and unidentified-gender seedlings that were established at the Plant and Food Research Site, Te Puke, New Zealand

			Total planted			QTL analysis			Transplant date		
Block	Date	# Planted	# ♀	# ♂	# Unidentified	# ♀	# ♂	# Unidentified	Winter 2015	Winter 2016	Not transplanted
3	2009-12-09	100	90	10	0	1	3	0	0	4	0
14 W	2010-03-03	13	11	0	2	5	2	0	0	7	0
35	2009-12-08	510	468	42	0	110	4	7	87	0	34
52	2010-03-03	545	439	10	96	130	11	5	0	129	17
Total		1168	1008	62	98	246	20	12	87	140	51

### Library preparation

For each individual, high molecular weight DNA was extracted from approximately 100 mg of leaf tissue using a standard cetyl trimethylammonium bromide (CTAB) protocol (Doyle 1987). The GBS method of Elshire *et al.* (2011) was used to obtain reduced representation of the genomes for the two parents and 278 individuals of their progeny selected for displaying the extremes of king flower per winter bud production (high or low) with a small number of individuals showing a median king flower production. GBS libraries were developed using the *Ape*KI restriction enzyme and set of 96 barcodes. Initial data were generated from GBS libraries from 96 individuals by Macrogen Ltd (https://dna.macrogen.com/eng/), and additional data were generated for these individuals as well as an additional 182 individuals by the Australian Genome Research Facility (http://www.agrf.org.au/). Samples were run on the Illumina HiSeq2000 (Illumina, San Diego, CA, USA) generating 100 bp single-end sequences.

### Sequencing data analysis

Data were split by barcode using fastx-multx [ea-utils/1.1.2-806 (Aronesty 2013)], where barcode sequences were also trimmed using additional settings (-d 1 -b -m 0 -q 20). Restriction enzyme sites were checked using fastq-multx. Adapter contamination was removed using trim_galore 0.4.3 (https://www.bioinformatics.babraham.ac.uk/projects/trim_galore/) with default settings but with “–stringency 5 –length 70.”

After barcode splitting and trimming, samples with less than 5,000,000 reads were excluded (one sample). Read counts ranged from 6,641,209 to 22,517,489, with a mean of 13,140,809 and median of 12,437,289; parents were sequenced in triplicate with depths varying from 11,824,577 to 16,011,240 (Table 2).

**Table 2:** Summary of the read count range, median, mean, for the male parent 28, the female parent ZE and the ZE28 progeny with the alignment rate, median, and mean

Group	Read count	Read count median	Read count mean	Alignment rate	Alignment median	Alignment mean
Parent 28	12933898–16011240	12933898	13771759	66.7–69.4	68.0	68.1
Parent ZE	11824577–14396284	12366343	12862401	70.1–84.5	82.2	79.0
All ZE28 progeny	6641209–22517490	12433689	12433690	52.7–93.8	75.1	76.0

Reads were aligned to a pre-release version of the *A. chinensis* reference Version 2 using “Red5” (PS1.1.68.5, DOI10.5281/zenodo.1297303) using BWA-MEM v0.7.15 (Gurevich *et al.* 2013). Alignment files were sorted and indexed with SAMtools 1.7 (Li *et al.* 2009) and Sample and Read Group IDs were appended to alignment files using Picardtools 2.10.1 (https://broadinstitute.github.io/picard/).

### Variant calling and filtering

Variants were called using Freebayes (Garrison and Marth 2012) with command line options “-p 6 -C 5 -k –min-mapping-quality 10 –genotype-qualities –use-mapping-quality.” Variants that were homozygous for one parent and heterozygous for the other parent and estimated to be in one single dose (simplex × nulliplex segregation) based on the allelic ratios in each sample were selected for further analysis. Raw variants were filtered using Freebayes based on the proportion of individuals successfully scored (call rate > 0.7) and maximum read depth (DP) of 30,000 (Supplementary Table S1).

### Linkage map construction

Linkage maps for the six homologs of both parents and 29 chromosomes were constructed using simplex × nulliplex markers and using the double-pseudo test cross strategy (Grattapaglia and Sederoff 1994) adapted to hexaploid. JoinMap^®^ 3.0 (https://www.kyazma.nl/) was used to construct the LGs. Grouping of loci was achieved with a minimum LOD (logarithm of the odds) score of 5 and regression mapping was used for map calculation using the Kosambi mapping function. A first draft of the linkage map was developed and then manually checked for the presence of double recombinants, due to genotypic errors. During that process, genotypic calls that were anomalous and would have misplaced markers compared to their expected location within the reference genome were manually corrected. The single nucleotide polymorphism (SNP) markers mapped onto the final linkage map were named according to their location in the reference *A. chinensis* genome assembly of “Red5” (PS1.69.0) (Pilkington *et al.* 2018). GBS marker locations based on the read mapping done on version PS1.68.5 were converted to physical locations in the assembly version PS1.69.0 using custom perl scripts.

### Collinearity of the genetic and physical maps and genome-wide recombination rates

The markers identified through the construction of the linkage map were arranged by their genetic distance (in cM) and physical distance (in Mb) in R version 1.2.5042 using the xyplot function in the lattice library, grouped by each homolog.

### Phenotypic assessment and QTL mapping

Flowering and fruit attributes were measured from the ZE28 seedlings in four different flowering/fruiting seasons (Supplementary Table S2). In the Southern Hemisphere, kiwifruit flowers mature in November with fruit harvest conducted in May. Season one encompassed flowering in November 2013 through fruit harvest in May 2014 and the storage fruit firmness assessments in August 2014. Season two data were collected from November 2014 through August 2015. November 2015 to August 2016 was defined as season three with season four being from November 2017 to August 2018.

In evaluating seasons one, two, three, and four, the total number of fruits per vine, the average fruit weight (total bulk fruit weight/number of fruit weighed), and the dry matter content were measured on all fruit-bearing vines. King flowers per winter bud and fruit firmness following 12 weeks of storage were measured in season four.

Fruit dry matter content was measured by cutting one equatorial slice of approximately 2 mm thickness and drying at 65° for 24 hours. The fruit dry matter content was calculated from the final dry weight and initial wet weight of the slices, recorded as a percentage of fresh weight. King flowers per winter bud were assessed in spring of the evaluation season as per the flowering protocol described by Snelgar *et al.* (1997). Fruit firmness, expressed as kilogram-force (kgf), was recorded 12 weeks postharvest using a GUSS penetrometer with a 7.9 mm diameter probe traveling at a downward speed of 5 mm/s. Penetrometer measurements were made on both the flat and round side of the fruit, at 90-degree angles, and then averaged.

For QTL detection, the traits were processed yearly (due to the climatic differences each season and the increase in the seedling maturity) and QTL detection with permutation tests were performed using MapQTL v5.0 (https://www.kyazma.nl/) and interval mapping. QTLs were declared significant if their LOD score was above 3.

### Data availability

Raw DNA-seq reads are available at NCBI under BioProject number PRJNA721532. Genotypic data for both parents are presented in Supplementary Table S1. The phenotypic data used for QTL detection are presented, in the same order at the genotypic data, in Supplementary Table S2. Supplementary Figure S1 contains the pedigree structure of the ZE28 family. Supplementary material is available at figshare: https://doi.org/10.25387/g3.13218716. The authors affirm that all data necessary for confirming the conclusions of the article are present within the article, figures, and tables.

## Results

### Generation of a bi-parental *A. deliciosa* cross

A large bi-parental cross containing 1168 seedlings that displayed a wide distribution of yield characters was used in this study (Table 3). Of particular interest was the variation of numbers of king flowers per winter bud. In the pedigree structure, the male parent was an accession from China, while the female was from the fourth generation of crossing from an accession originally imported into New Zealand. Both the female and male parents had inbreeding levels of 0 and so were unrelated to each other (coefficient of ancestry = 0). From this population, 278 individuals were selected for genotyping based on the extremes (high and low) king flower production.

**Table 3:** Population averages for vine yield [fruit number, king flowers per winter bud, fruit weight (g)] and fruit quality [dry matter content (%), fruit firmness 12 weeks postharvest (kgf)] traits in the hexaploid ZE28 population that had QTL analysis, compared to *Actinidia chinensis* var. *deliciosa* “Hayward”

Trait	Evaluation season	ZE28 n	Average	Range	Standard deviation	“Hayward” n	“Hayward” average	“Hayward” range	“Hayward” standard deviation
Fruit number	1	245	2.5	0–61	5.8	20	18.6	2–68	17.7
Fruit number	2	245	20.3	0–178	25.4	8	14.5	1–27	8.3
Fruit number	3	245	39.6	0–250	53.5	14	6.7	1–33	1.5
Fruit number	4	221	30.6	0–130	27.6	8	15.6	4–42	14.5
King flowers per winter bud	4	221	1.65	0–4.68	1.04	8	1.23	0.45–2.36	0.73
Fruit weight (g)	1	134	55.4	15.9–109.9	16.9	20	92.6	65.3–108.1	9.5
Fruit weight (g)	2	146	72.2	42.4–124.9	14.8	8	99.2	74.4–120.5	13.9
Fruit weight (g)	3	125	74.1	28.9–115.4	16.8	14	97.9	72.2–127.5	18.5
Fruit weight (g)	4	204	72.6	72.6–115.5	16.1	8	108.4	101.0–117.6	5.9
Dry matter content (%)	1	133	18.3	13.5–23.8	1.8	14	17.2	14.6–18.9	1.0
Dry matter content (%)	2	136	19.9	14.7–24.5	1.7	5	17.8	17.1–18.3	0.4
Dry matter content (%)	3	127	18.5	13.4–24.3	1.9	15	17.2	15.1–19.3	0.4
Fruit firmness, 12 weeks PH (kgf)	4	204	0.83	0.23–2.1	0.34	2	1.38	1.04–1.73	0.48

### Sequencing data, SNP detection and filtering

**Figure 1:**
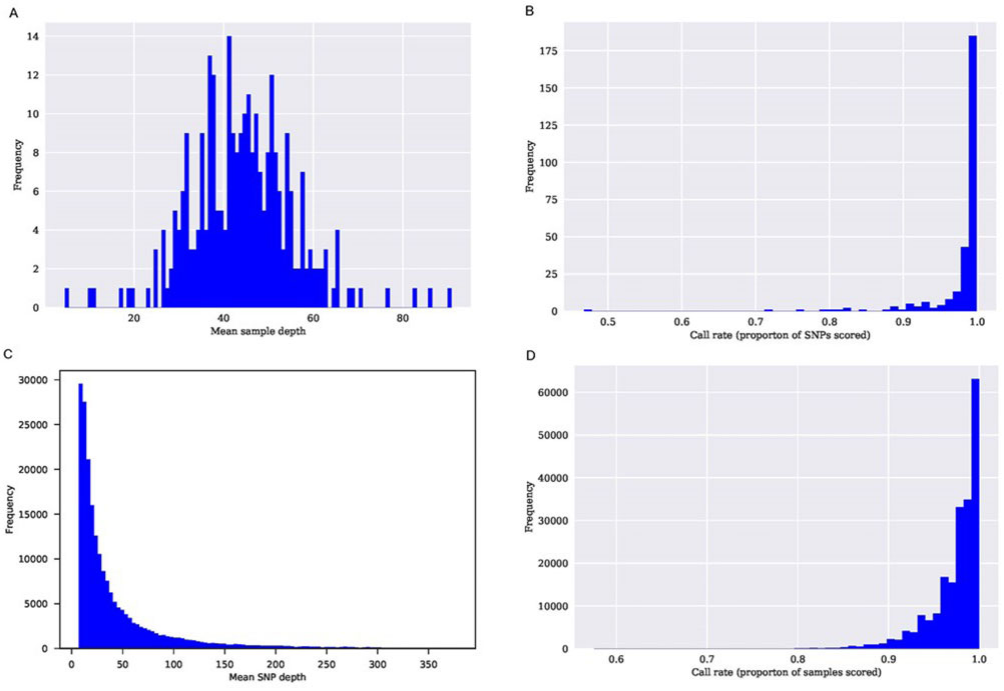
Statistics of SNP markers obtained by GBS in the hexaploid ZE28 kiwifruit segregating population; (A) frequency of mean sample depth, (B) frequency of SNP call rate as a proportion of SNPs recorded, (C) frequency of mean SNP read depth, (D) frequency of call rate as a proportion of the samples scored.

To select an appropriate restriction enzyme that provides a balance between coverage depth and fragment length, an in silico digestion of the kiwifruit genome was modeled for 5 candidate restriction enzymes (Supplementary Figure S2). The *Ape*KI restriction enzyme was chosen for DNA fragmentation as it provided a good spread of fragments and was methylation sensitive reducing the frequency of repetitive DNA represented (Hilario *et al.* 2015). In total, 3.5 billion raw sequencing reads were obtained from GBS libraries of triplicates of both parents and the 278 selected segregating full-sib individuals, and 74.6% of sequencing reads successfully aligned to the reference genome assembly of *A. chinensis* (Table 4). Ten sets of reads from the full sibling seedlings were ultimately removed due to low read counts and poor mapping (<50%), providing genotyping for 268 individuals. The estimated mean and median mapping rate for the remaining population was 76% and 75%, ranging from 53 to 94%. A total of 435,901 variants were detected. The median of the mean depth for each sample was distributed around 40X (Figure 1A). Both parents had higher read depth (138X and 127X for the female and male parent, respectively) due to three replicates being sequenced and merged. The call rate for SNPs, measured as the proportion of SNPs being successfully scored in the progeny and parents, was distributed over 90% (Figure 1B). The mean SNP depth was mostly consistent with a median value of 44.2 (Figure 1C) and the call rate, calculated as the proportion of sample being successfully scored, had a median of 0.99 (Figure 1D).

**Table 4:** Summary of the number of homologs identified per chromosome and the percentage of marker coverage across all chromosome homologs identified in the linkage maps for the female and male hexaploid kiwifruit parents

	Female		Male	
Chromosome	Number of homologs	% coverage	Number of homologs	% coverage
1	6	67.59	6	84.98
2	6	88.29	6	85.29
3	7	50.22	6	87.08
4	6	74.72	6	85.32
5	7	59.63	6	56.89
6	6	71.92	6	87.70
7	6	65.73	6	81.85
8	8	72.79	6	90.06
9	6	79.36	7	74.12
10	4	92.08	6	92.66
11	6	81.83	6	86.56
12	6	60.09	7	74.92
13	6	86.09	6	93.55
14	7	43.45	6	71.66
15	7	42.39	6	79.29
16	6	44.59	6	71.98
17	6	63.87	6	72.48
18	7	62.49	6	59.60
19	8	83.70	6	84.61
20	6	53.62	6	78.55
21	6	71.12	6	76.11
22	6	34.38	6	59.11
23	6	68.13	6	61.28
24	8	38.71	6	59.22
25	6	44.45	6	54.92
26	5	45.85	7	46.99
27	9	37.68	5	61.68
28	4	71.63	6	81.34
29	6	90.09	6	81.74
Total	183	63.67	176	75.23

### Linkage map construction

**Figure 2:**
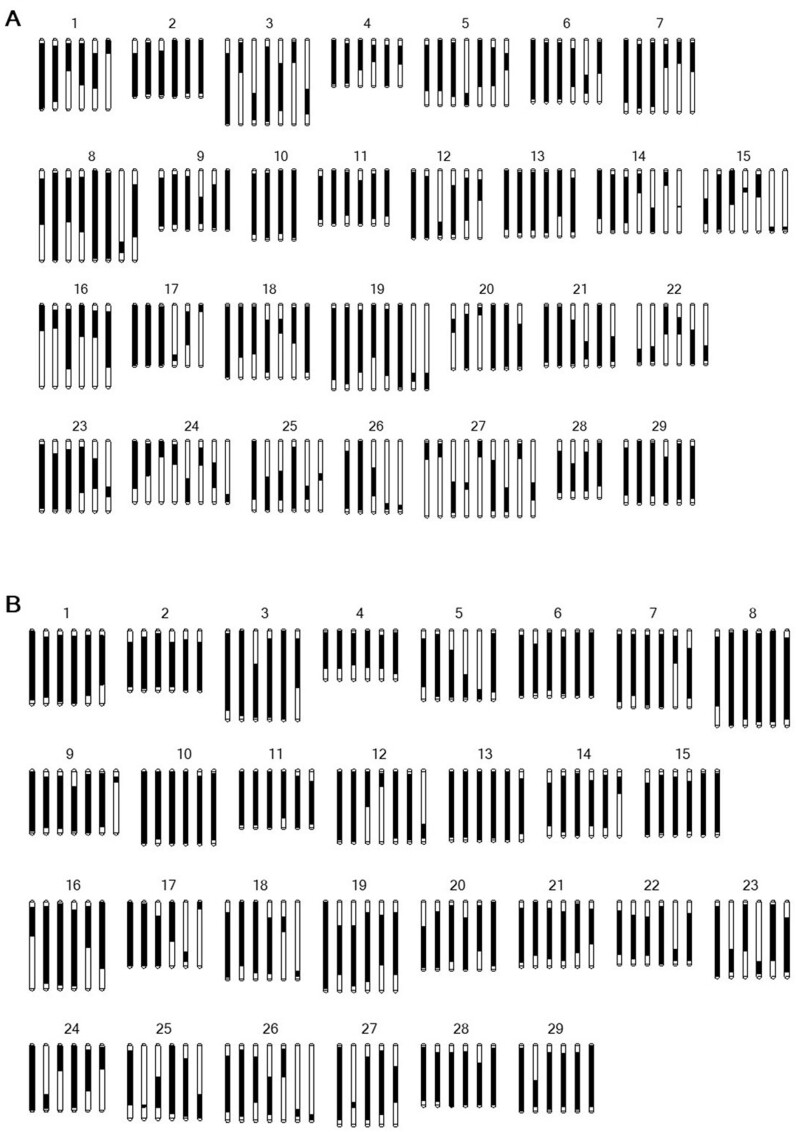
Schematic of the marker coverage in the linkage map for the chromosome homologs from the ZE female and 28 male parents comprising the ZE28 segregating seedling population.

**Figure 3:**
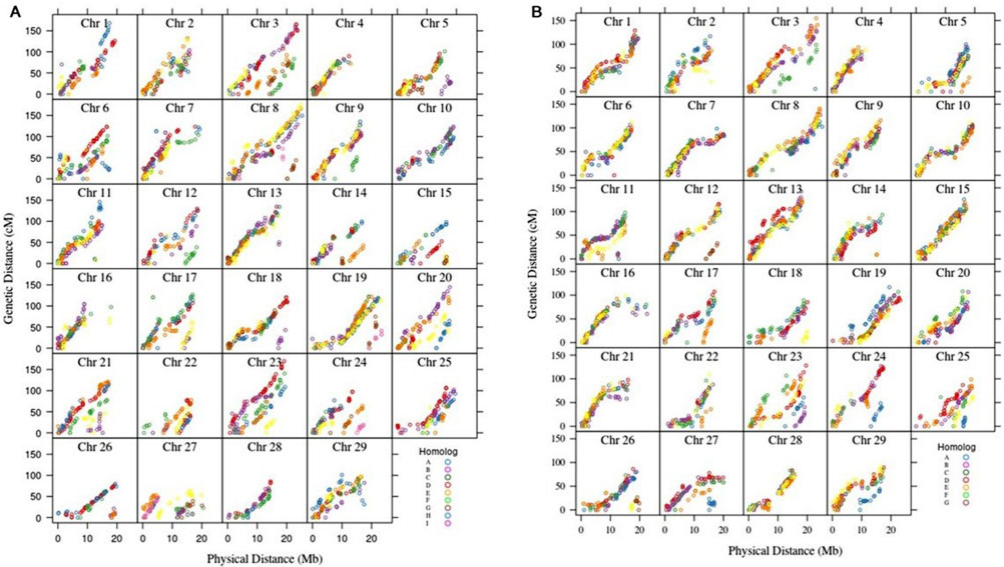
Alignment of the genetic and physical maps obtained from the hexaploid kiwifruit genotyping by sequence (GBS) dataset. (A) ZE female and (B) 28 male parent. Each dot represents the genetic distance (cM) and physical distances (Mb) for an identified marker across the 29 chromosomes. The different homologs for each chromosome are represented by a unique color within each chromosome panel. The slope of the homolog is representative of the recombination rates.

SNPs that were present in the parents in a simplex × nulliplex mode (*e.g.*, AAAAAG × AAAAAA), and reciprocal, were selected for the linkage map construction. Many genotyping errors were found in the data and visible as suspicious double recombinants occurring between markers positioned at small physical distance on the reference genome. After a manual correction of these errors, there were 3686 and 3940 mapped SNP markers across 183 and 176 LGs in the female and male parents, respectively (Table 4, Figure 2 and Supplemental Figure S3, A and B). In the female ZE parent, the map spanned a total of 14,957 cM, with an average of 81.7 cM per LG, an average marker distance of 4.6 cM, and a maximum distance between markers of 16.5 cM. For the male 28 parents, the map spanned a total of 10,869 cM with an average LG length of 107.6 cM. The average distance between the male parent markers was 5.8 cM, with the largest gap being 39 cM. The marker content and order along the linkage groups (LGs) were consistent with the Red5 genome assembly of *A. chinensis*, as demonstrated by the high collinearity between the reference genome and the LGs (Figure 3). On average, the female and male linkage maps covered respectively 63.6% and 75.2% of the genome assembly. The male linkage map was more saturated than the female map, with 25 out of the 29 chromosomes having six LGs, corresponding to the six expected homologs, and 119 out of 176 LGs (67.6%) covering greater than 75% of the chromosome physical length based on the Red5 assembly (PS1.69.0). In the female map, 17 of the 29 chromosomes had six homologs, and 89 out of 183 LGs (48.6%) covered more than 75% of the chromosome length (Figure 2).

### Fruit and yield phenotypes

**Figure 4:**
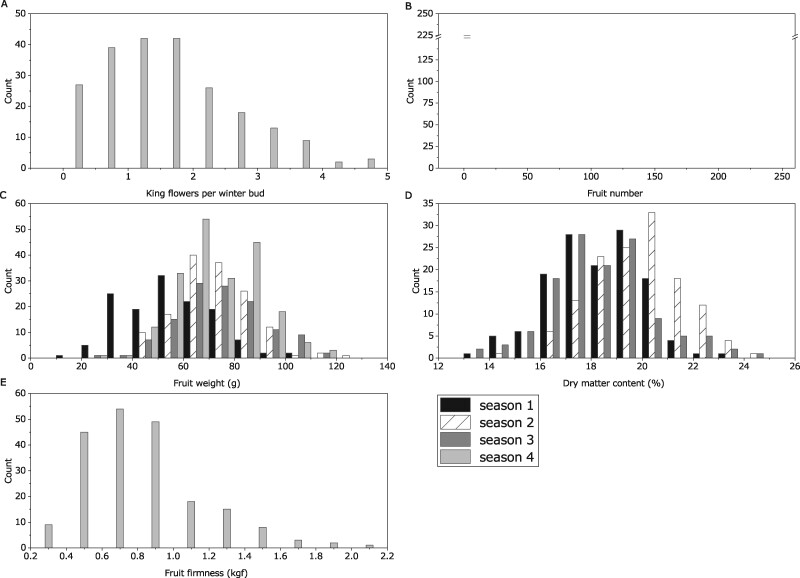
Trait distribution for (A) king flowers per winter bud, (B) fruit number, (C) fruit weight (g), (D) dry matter content (%), and (E) fruit firmness (kgf) across the four evaluation seasons in the ZE28 segregating population.

The industry standard cv “Hayward,” of comparable age, were grown in the same block locations as the ZE28 progeny to act as a reference. Key phenotypic traits including the number of king flowers per winter bud, fruit number and weight, and size were measured over 1–4 seasons (Table 3 and Figure 4). As the ZE28 seedlings matured, there was an increase in the number of fruit produced as well as the average fruit weight each year. On average, “Hayward” produced less fruit than the ZE28 seedling population average, 13.8 *vs* 23.2 fruit per vine. However, fruit weight was higher for “Hayward,” 99.5 *vs* 68.6 g, respectively. The dry matter content of “Hayward” was in the lower quartile of the seedling distribution for all years assessed, 17.4 *vs* 18.8% dry matter content. While the distribution of the ZE28 seedlings was wider than that of “Hayward” for fruit firmness 12-weeks post-harvest, the average of “Hayward” was higher, 0.83 *vs* 1.38 kgf. The distribution of numbers of king flowers per winter bud ranged from 0.45 to 2.36 in Hayward with a mean of 1.23. The selected genotyped population showed a spread of 0 to 4.68 king flowers with an average of 1.68.

### QTL detection

Nine QTLs were identified with LOD scores greater than 3.0 across the four evaluation seasons, two from the female parent and seven from the male parent, for the following traits: the number of king flowers per winter bud, fruit number, fruit weight (g), dry matter content (%), fruit firmness 12-weeks post-harvest (Table 5). QTLs were inherited from both the female and male. The variance explained for these QTLs ranged between 5.6 and 14.5%. Permutation tests conducted on the QTLs indicated two were strong and the others were suggestive.

**Table 5:** Quantitative trait loci for yield and fruit traits in the hexaploid ZE28 mapping population

Trait	Evaluation season	Linkage group	Closest marker	Parent	LOD	Permutation (95% threshold)	Percent variance explained	KW	**KW significance levels** ^a^
Fruit number	3	6D	LG6:2142758	28	3.45	3.9	6.4	9.1	****
Fruit number	3	20C	LG20:15935274	28	3.10	3.9	5.8	10.04	****
Fruit number	3	5C	LG5:5184121	28	3.02	3.9	5.6	16.52	*******
King flowers per winter bud	4	20E	LG20:11756985	28	3.26	4.0	6.5	11.2	*****
Fruit weight (g)	1	5F	LG5:3333511	ZE	3.17	4.0	9.5	14.33	******
Fruit weight (g)	1	13E	LG13:74766	28	3.15	3.8	9.5	13.08	******
Dry matter content (%)	1	24A	LG24:16322191	28	3.21	3.9	10.3	14.74	******
Dry matter content (%)	3	6D	LG6:12592191	ZE	4.32	3.6	14.5	16.32	*******
Fruit firmness, 12-weeks postharvest (Kgf)	4	15B	LG22:13315746	28	4.38	3.8	9.4	12.74	******

Traits include fruit number count, king flowers per winter bud, fruit weight (g), dry matter content (%), and fruit firmness measured 12-week postharvest (kgf). Year is the year of data collections, LG is the chromosome homolog where the quantitative trait locus (QTL) is located, closest marker denotes the marker location nearest to the QTL based on the Pilkington *et al.* publication (Pilkington *et al.* 2018), parent identifies the male parent 28 or the female parent ZE, LOD is the “logarithm of the odds” for the presence of a QTL at a particular location, the permutation (95% threshold) indicates the LOD when accounting for false positives, percent variance explained is the contribution the QTL makes to the trait, KW is the Kruskal-Wallis one-way ANOVA on ranks.

a KW significance levels: *<0.1, **<0.05, ***<0.01, ****<0.005, *****<0.001, ******<0.0005, *******<0.0001.

## Discussion

The study demonstrates that it is possible to identify QTLs in a hexaploid population using a GBS approach. QTLs for king flowers per winter bud, dry matter content, fruit size, fruit number, and fruit firmness were discovered. These are the first QTLs to be reported in hexaploid kiwifruit. QTL detection was possible because the progeny size was sufficient for such analysis. It is well known that the ability to detect significant QTLs is based, among other things, on the population size (Beavis *et al.* 1994). The population size in previously published SNP-based diploid kiwifruit genetic linkage maps ranged from 94 individuals (Scaglione *et al.* 2015) to 230 individuals (Zhang *et al.* 2015; Tahir *et al.* 2019). Such small population sizes may inflate the estimated QTL effect due to the Beavis effect (Xu 2003). Indeed Scaglione *et al.* (2015) recognized the challenge of using a small progeny size of only 47 female vines when reporting a QTL for fruit size on chromosome 6 explaining 41.8% of the variance. Beavis *et al.* (1994) illustrated how mapping studies with 100 individuals were subjected to greatly inflated genetic estimates of QTL effect sizes. Our study using 278 individuals provided QTLs with a lower explained variance, which is probably better estimated than studies using smaller progeny sizes. Yield attributes and their associated components (plant height, branching types, root structures, and so on) are known to be highly polygenic traits influenced by the environment. This has been illustrated in multiple crops, with yield-associated QTLs previously published in tomato (Gur *et al.* 2011), wheat (Maccaferri *et al.* 2008), barley (Von Korff *et al.* 2008), and brassica (Shi *et al.* 2009). The fact that this linkage map was able to identify QTLs for highly polygenetic traits (yield and fruit firmness) illustrates its usefulness.

GBS, as a process, will have errors introduced at each step in the process, both in the laboratory as well as in the data processing. Challenges exist between differentiating what is an error and what is an accurate sequence read. The most common errors in reading the GBS data are missing genotype information due to low sequence read depth (Bilton *et al.* 2018) and making the distinction between a real double crossover event or a false data point (Cartwright *et al.* 2007). A manual process was decided to be appropriate to review the presence of double cross-over events by taking into account the physical distances between markers. The removal of genotyping errors masked as double crossover events improved the quality of the linkage map. However, in comparison to other map construction software (Bilton *et al.* 2018), JoinMap typically results in an expanded genetic linkage map as well as some incorrect marker identification/order.

A linkage map was built for both parents of a hexaploid *A. chinensis* var. *deliciosa* population, using GBS and SNP markers segregating in a simplex × nulliplex fashion. This is the first map for hexaploid kiwifruit and a new addition to maps for polyploid Ericaceae species, as a linkage map of tetraploid blueberry was published (McCallum *et al.* 2016). Our linkage map produced a similar density of markers compared to what is reported in previous SNP and SSR-based maps of diploid kiwifruit (Liu *et al.* 2017) based on the average number of markers per LG (Testolin *et al.* 2001; Fraser *et al.* 2009; Scaglione *et al.* 2015). As this experiment was a hexaploid progeny and simplex × nulliplex markers were used for building LGs corresponding to homologs, it was encouraging that markers were placed across all 29 chromosomes for each parent. In total, 183 and 176 LGs were built for the female and male parent, respectively. Theoretically, 174 (29 chromosomes × 6 homologs) LGs were expected. The difference between the expected and obtained LG numbers can be attributed to the number of generations of selection in the parents and the inability to get full map saturation along each homolog. The female parent ZE has had more selection pressure than the male parent 28 and has a larger number of LGs, including some chromosomes for which up to nine LGs were built. The nine LGs may be a result of incomplete coverage of a single homolog resulting in two homologs of unknown order.

The plots of the genetic and physical maps (Figure 3) indicate the markers placed on the physical map were effective. An acceptable plot is where the markers are defined by a monotonically increasing function. Most of the homologs fit the monotonically increasing function, but there were exceptions on the distal ends of chromosome 6B, 8A, 21D, and 29B for the ZE parent and all homologs of chromosome 16, and 2F for parent 28. The slope of the plots is indicative of the recombination rates (Rezvoy *et al.* 2007). Outside the centromere location, which typically has lower rates of recombination, the recombination rates were appropriate for most homologs.

The strategy to use simplex × nulliplex markers may explain the lack of full saturation for the ZE map as it may not be possible to find such markers segregating for homologs that are fixed. A potential solution for saturating the map would be to include other types of marker segregation, such as duplex × multiplex, simple × × simplex, and higher dosage ratios. However, it was not possible to achieve this using this dataset because the read depth obtained (40X) was insufficient to accurately estimate the allelic dosages in the progeny. As the linkage map is based on SNP markers aligned to the reference genome of kiwifruit, it was possible to obtain an indication of how much of the genome was covered by markers. Again, the female ZE map is less saturated than the male 28 maps, possibly because of a higher selection pressure or higher homozygosity between homologs in the female. The male linkage map is well saturated with only four chromosomes for which the number of LGs is not equal to six. Future directions would include integrating the dosage effects as reported in chrysanthemum and potato (Hackett *et al.* 2013; van Geest *et al.* 2017) by using other genotyping technologies such as SNP arrays or hybridization-based sequencing technology. These technologies are capable of more accurate allelic dosage estimation which is crucial for using polyploid-aware software for genetic linkage analysis, such as polyMapR (Bourke *et al.* 2018).

## Conclusions

Green kiwifruit production has been dominated by the “Hayward” cultivar in the last 60 years. It is important for kiwifruit breeders to use modern molecular tools to aid the selection of superior hexaploid kiwifruit cultivars for consumer traits such as taste, dry matter content, storability and size, and production traits such as yield and tolerance to *Pseudomonas syringae* pv. *actinidiae*. Furthermore, the mapping of genetic loci linked to these traits and alignment with the genome of kiwifruit will enable researchers to shed new light on the control of these traits.
